# Natural Course of Asymptomatic Walled-off Necrosis due to Acute Pancreatitis

**DOI:** 10.5152/tjg.2026.24348

**Published:** 2026-01-15

**Authors:** Melike Bektaş, Şencan Acar, Ahmet Tarık Eminler, Mukaddes Tozlu, Bilal Toka, Fuldem Mutlu, Mustafa İhsan Uslan, Erkan Parlak, Aydın Şeref Köksal

**Affiliations:** 1 Department of Internal Medicine, Sakarya University Faculty of Medicine, Sakarya, Türkiye; 2 Department of Gastroenterology, Sakarya University Faculty of Medicine, Sakarya, Türkiye; 3 Department of Radiology, Sakarya University Faculty of Medicine, Sakarya, Türkiye; 4 Department of Gastroenterology, Hacettepe University Faculty of Medicine, Ankara, Türkiye

**Keywords:** Acute pancreatitis, intervention, natural course, WON

## Abstract

**Background/Aims:**

Walled-off necrosis (WON) is a well-developed, enclosed accumulation of fluid, comprising a mixture of solid and liquid necrotic material, located within the pancreas and its surrounding area. Managing patients with asymptomatic WON is controversial due to limited information available about its natural history. The study aimed to determine the long-term natural course of WON in acute pancreatitis (AP) patients who remained asymptomatic and did not undergo any interventional treatment upon initial diagnosis.

**Materials and Methods:**

Acute pancreatitis patients who were followed between June 2016 and December 2019 were retrospectively evaluated. Among them, patients who developed WON after the AP attack and did not undergo any therapeutic intervention upon initial diagnosis due to their asymptomatic course were enrolled in the study.

**Results:**

The asymptomatic WON patients (n = 31) were followed up for a mean duration of 25.6 ± 17.3 months. During the follow-up, 10 (32.3%) patients required interventional treatment within an average of 124.9 ± 189.5 days. Two patients died while under asymptomatic follow-up. In 12 patients (38.7%) WON disappeared; and in 7 (22.5 %) patients, mean size of the largest WON decreased from 64.7 ± 34.2 to 29.8 ± 27.4 mm. The extent of necrosis and the initial WON size were significantly higher in those requiring interventional treatment [AUC (area under the curve) were 0.844 and 0.733, respectively].

**Conclusion:**

The management of WON usually includes close monitoring of the patient. The initial characteristics of WON (e.g., extent of necrosis, size, etc.) may aid in determining the necessity for drainage during the follow up of asymptomatic patients.

**Main Points**

- Walled-off necrosis (WON) usually develops ≥4 weeks after the acute onset of necrotizing pancreatitis and is estimated to have a prevalence of 1%-9% among patients diagnosed sil with acute pancreatitis (AP).
- Data about the natural course of asymptomatic WON is limited and there is scant evidence to support the conservative management of asymptomatic WON, as these recommendations are largely based on expert opinion.
- The present study aimed to determine the long-term natural course of WON in AP patients who remained asymptomatic and did not undergo any interventional treatment at the time of diagnosis [n: 31, mean follow-up duration of 35.6 ± 10.2 (range: 17-51) months].
- Remove the study showed among the patients with WON who were asymptomatic at diagnosis, the majority of them sil (67.7%) remained asymptomatic during the follow-up period and did not require any interventional treatment related to WON.

## Introduction

The majority of acute pancreatitis (AP) cases present in a mild and self-limited form. However, approximately 20% of patients develop moderate to severe disease involving local pancreatic complications, systemic organ dysfunction(s), or a combination of both.^1^ Severe AP has a significantly higher mortality rate of 15%-30%, compared to the 0%-1% mortality rate associated with mild AP.^2^ Local complications include acute peripancreatic fluid collection, acute necrotic collection (ANC), pancreatic pseudocyst, and walled-off necrosis (WON).^3^

Walled-off necrosis is a well-developed, enclosed accumulation of fluid, comprising a mixture of solid and liquid necrotic material, located within the pancreas and its surrounding area. It is characterized by an established inflammatory wall.^4^ WON usually develops ≥4 weeks after the acute onset of necrotizing pancreatitis and is estimated to have a prevalence of 1%-9% among patients diagnosed with AP.^5^^,^^6^ It may lead to symptoms such as abdominal pain, early satiety, fever, jaundice, or gastric outlet obstruction, or it may remain asymptomatic. Symptomatic WON is an indication for intervention, which may include endoscopic, radiologic, or surgical approaches.^7^ However, managing patients with asymptomatic WON is controversial due to limited information available about its natural history. Approximately half of the WON cases (37%-59%) undergo spontaneous regression.^8^ Regardless of the size of the WON or the cause of AP, current recommendations suggest conservative therapy for asymptomatic WON patients.^3^^,^^9^ However, the conservative approach carries a risk of fatal outcomes, which may include infection, bleeding, and rupture. Furthermore, there is scant evidence to support the conservative management of asymptomatic WON, as these recommendations are largely based on expert opinion. The studies available on the natural history of asymptomatic WON are retrospective and include small sample sizes (16-47 patients), diverse study populations, and relatively short follow-up periods.^10^^,^^11^

Due to the limited evidence in the literature, the present study aimed to determine the long-term natural course of WON in AP patients who remained asymptomatic and did not undergo any interventional treatment at the time of initial diagnosis.

## Materials and Methods

Acute pancreatitis patients who were followed at the Sakarya University Faculty of Medicine Department of Gastroenterology between June 2016 and December 2019 were retrospectively evaluated. Among them, patients who developed WON after the AP attack and did not undergo any therapeutic intervention at initial diagnosis due to their asymptomatic course were enrolled in the study. Data were collected by reviewing medical records at the relevant clinic and through phone contact with the patients. Patients who were initially symptomatic and underwent endoscopic, percutaneous, or surgical interventions, those with chronic pancreatitis, and those who did not attend regular follow-up visits for at least 6 months and could not be reached by phone were excluded from the study.

Demographic characteristics of the patients, etiology, and severity of AP were documented. The baseline characteristics of WON, including the number of lesions, size, localization (pancreatic, peripancreatic, and mixed), wall thickness, and the extent of necrosis (%) at the diagnosis of AP, as well as the presence of associated disconnected pancreatic duct, were evaluated by the same radiologist. Changes in size during follow-up imaging assessments (at diagnosis, third-month follow-up, sixth-month follow-up, and the last imaging) were recorded. Patients whose WON did not resolve on follow-up imaging but who did not attend the control visit(s) were contacted by phone, and clinical information was obtained. Additionally, new imaging was offered to those who did not undergo any therapeutic intervention. The endpoint of the study was defined as the final control date, the onset of symptomatic WON requiring intervention, or the complete resolution of WON. The WON patients who became symptomatic and/or developed complications during the follow-up were documented. Any procedures performed on these patients during the follow-up period were identified. The therapeutic interventions were determined by the consensus of gastroenterologists and surgeons. The definitions were based on the revised Atlanta classification.^4^

### Ethics Statement

The study was conducted in accordance with the ethical principles of the Declaration of Helsinki. Ethical approval was obtained from the local ethics committee of Sakarya University Fculty of Medicine on October 20, 2020 (71522473/050.01.04). As this study was designed as a retrospective review of patient records and imaging data, individual informed consent was not required or obtained. All patient information was anonymized prior to analysis to ensure confidentiality and data protection.

### Outcomes of the Study

The primary outcome of the study was to determine the frequency of the need for interventional treatment during the follow-up period in asymptomatic WON patients who did not initially require intervention. The secondary outcome was to identify the baseline clinical and radiologic characteristics that contribute to the need for interventional treatment during the follow-up period.

### Statistical Analysis

Data were analyzed using SPSS 21.0 software (IBM SPSS Corp.; Armonk, NY, USA) . Results were given as mean ± SD (standard deviation). Pearson chi-square and Fisher exact test were used to compare categorical data. Student’s *t*-test and Mann–Whitney *U*-test analysis of variance were used to compare numerical data. The predictive value of WON characteristics for the need for therapeutic intervention was assessed by receiver operating characteristic (ROC) analyses. The ROC analyses were determined by area under the curve (AUC) and 95%. After ROC analyses, the threshold values with the best diagnostic performance were obtained with the “Youden index.” Sensitivity and specificity of these thresholds were calculated. A *P* value <.05 was considered statistically significant.

## Results

### Baseline Characteristics of the Study Population

**Figure 1.**
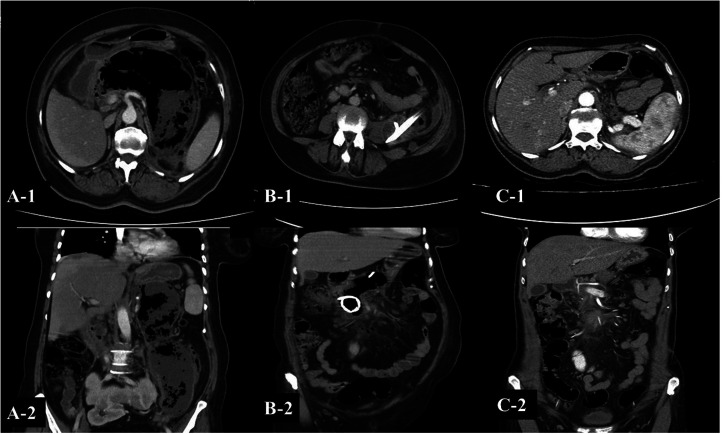
A forty-nine-year-old female patient. (A 1-2). An infected WON extending from the pancreas to the pelvis with air images inside. (B 1-2) Two percutaneous drainage catheters were inserted. (C 1-2) Complete resolution of WON was observed on follow-up CT scan 4 months after the procedure.

A total of 1173 patients with AP were followed throughout the study period. Out of these patients, 46 developed WON and were subsequently enrolled in the study. The mean age of the patients was 58.2 ± 13.7 years, with 26 individuals (56.5%) being male. Out of the 46 patients with WON, 4 were excluded from the study due to insufficient follow-up data. Eleven patients had indications for drainage during the initial admission because of the presence of symptoms, mostly due to abdominal pain and fever (55.5%). Four patients died before the intervention: 1 due to sepsis associated with WON, 1 due to pneumonia, 1 due to associated comorbidities including a cerebrovascular accident, and 1 due to unknown causes. The remaining 7 patients underwent drainage (3 endoscopically, 2 percutaneously, and 2 surgically) (Figure 1). The baseline demographic, clinical, and radiologic features, as well as the therapeutic interventions, of all patients with WON are outlined in Table 1.

The remaining 31 asymptomatic patients constituted the study group. The mean age of the patients was 59.3 ± 14.2 years, and 17 (54.8%) were male. The etiology of AP was biliary in 19 (61.3%) patients, and 26 (83.9%) had moderately severe AP. While the number of WON was 1 in 16 (51.6%) patients, it was 2 in 10, 3 in 4, and 4 in 1 patient. WON was localized in the pancreas in 24 (77.4%) patients, while 6 (19.4%) were extra-pancreatic, and 1 was combined. WON was extensively distributed, covering the entire pancreas in 18 (75%) patients with pancreatic localization. The mean size of the largest WON was 101.1 ± 48.0 mm. The mean extent of necrosis within the largest WON was 57.1 ± 24.0% and the mean WON wall thickness was 2.8 ± 0.9 mm (Table 2). Disconnected pancreatic duct syndrome was observed in 2 patients.

### The Clinical Course of the Asymptomatic WON Patients

**Figure 2.**
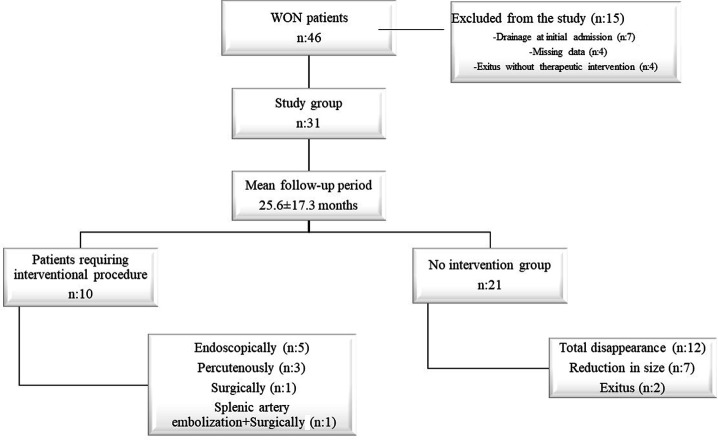
The clinical course of patients with WON.

**Figure 3.**
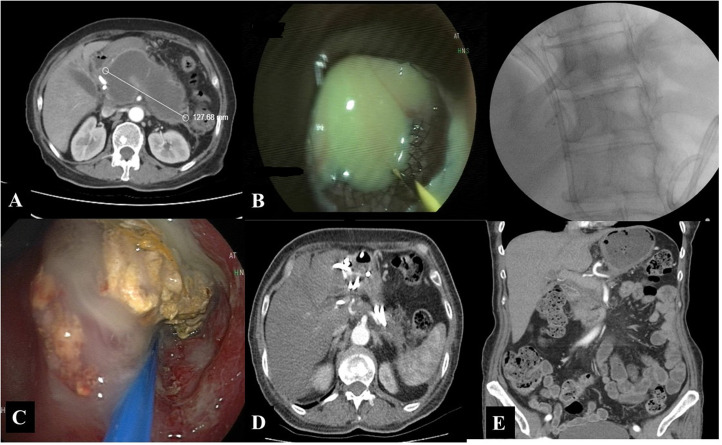
A 68-year-old female patient. A) A 12 cm-sized WON along the pancreas. B) Placement of a LAMS and a nasocystic catheter into the WON cavity using an endoscopic approach. C) Endoscopic necrosectomy. D) Follow-up image showing pigtail pigtail plastic stents 2 months post-procedure. E) Complete resolution of WON seen on a follow-up CT scan 4 months after the procedure.

**Figure 4.**
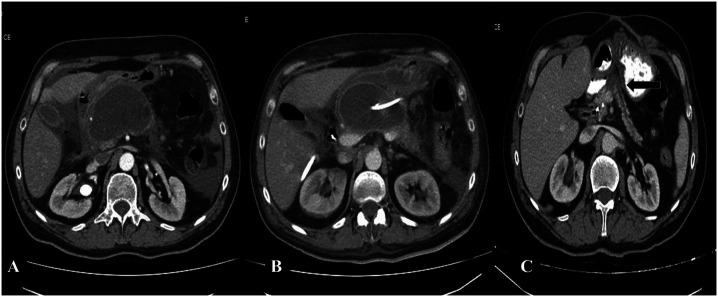
A 57-year-old male patient. A) A 10 cm-sized WON in the pancreatic head-body segment. B) Initial percutaneous intervention due to abscess symptoms abscess symptoms. C) Surgical necrosectomy was performed after 2 weeks because of no response to percutaneous intervention. Patient developed cystocutaneous fistula with atrophy and dilated duct in the pancreatic tail seen on CT imaging (disconnected pancreatic duct) (Black arrow).

The asymptomatic WON patients (n = 31) were followed up for a mean duration of 25.6 ± 17.3 months (range: 1-51). During the follow-up, 10 (32.3%) patients required interventional treatment within an average of 124.9 ± 189.5 (range: 13-615) days (Figure 2). The time to the requirement for interventional treatment was <3 months in 6 patients, between 3 and 6 months in 2 patients, between 6 and 12 months in 1 patient, and >12 months in 1 patient. The interventions included WON drainage in 9 patients and embolization of the splenic artery pseudoaneurysm in 1 patient. The reasons for WON drainage were the development of compression symptoms (abdominal pain, nausea, and vomiting) in 6 patients and infected WON (fever, abdominal pain, elevated C-reactive protein (CRP) levels, and/or the presence of extraluminal gas in the pancreatic/peripancreatic region on computed tomography (CT) in 4 patients. Drainage was performed endoscopically in 5 patients (Figure 3) (one of whom had a spontaneous fistula to the duodenum), percutaneously in 3 patients, surgically in 1 patient. The patient who underwent splenic artery embolization due to pseudoaneurysm underwent surgical cystogastrostomy in the follow-up (Table 3). However, 1 patient in the percutaneous treatment group underwent surgical intervention following percutaneous drainage and died due to sepsis and multiorgan dysfunction on the postoperative day 93. Another patient in the percutaneous treatment group underwent surgical necrosectomy 17 days after the percutaneous drainage due to inadequate recovery and is currently doing well (Figure 4). Finally, 1 patient in the surgery group underwent percutaneous drainage due to an extra-pancreatic collection that did not regress in size during follow-up. She recovered after the second procedure, and the WON completely disappeared at the second month follow-up imaging.

**Figure 5.**
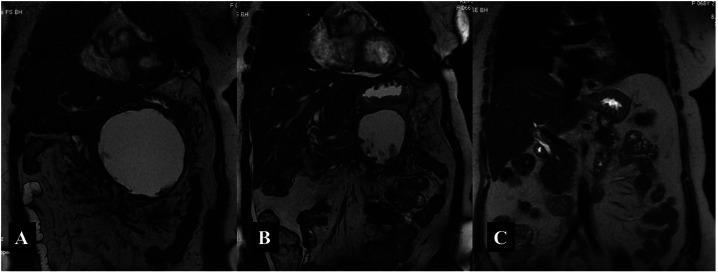
A 66-year old asymptomatic female. A) An 11 cm-sized WON in the midline of the abdomen. B) Reduction of WON size to 7 cm on MRI after 5 months. C) Complete resolution of WON observed on MRI obtained after 9 months.

Twenty-one patients (67.7%) remained asymptomatic and did not require any interventional treatment related to WON during a mean follow-up duration of 35.6 ± 10.2 (range: 17-51) months. Two patients died while under asymptomatic follow-up: 1 due to a myocardial infarction and the other patient due to COVID-19 (9.5%). In 12 patients (38.7%) WON disappeared (Figure 5); and in 7 (22.5 %) patients, mean size of the largest WON decreased from 64.7 ± 34.2 to 29.8 ± 27.4 mm.

### Factors Predicting the Need for Therapeutic Intervention

**Figure 6.**
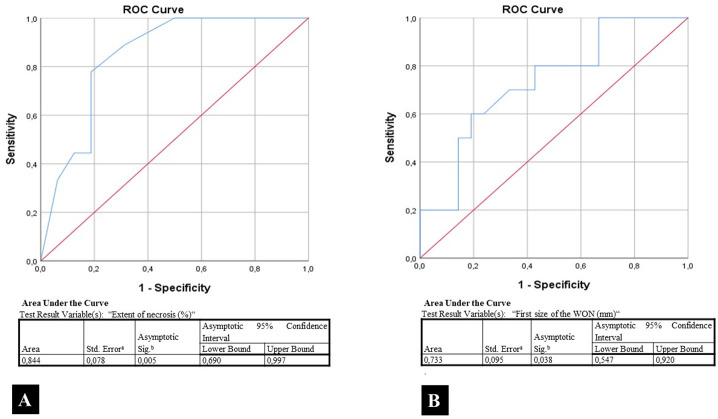
The AUC (area under the curve) for the extent of necrosis and initial size of WON. A) Extent of necrosis (%) B) Size of WON (mm).

There was no statistically significant difference between the mean age, gender distribution, severity of AP, number, location, wall thickness of WON in the groups requiring interventional treatment or not. However, the extent of necrosis and the initial WON size were significantly higher in those requiring interventional treatment. Additionally, biliary etiology was significantly more common in those requiring intervention (Table 4). The AUC for the extent of necrosis and initial WON size were 0.844 and 0.733, respectively (Figure 6). The ROC curve analysis indicated that the optimal extent of necrosis cut-off point for the requirement of interventional treatment was 55%, yielding sensitivity and specificity values of 88.9% and 68.7%, respectively. The optimal initial WON size cut-off point for the requirement of interventional treatment was 98.5 mm, yielding sensitivity and specificity values of 88.9% and 62.5%, respectively.

## Discussion

In the current study, it was demonstrated that among the patients with WON who were asymptomatic upon diagnosis, the majority of them (67.7%) remained asymptomatic during the follow-up period and did not require any interventional treatment related to WON.

Acute pancreatitis is characterized by varying degrees of severity, with the majority of patients experiencing a mild episode that recovers without serious complications. Nevertheless, a significant proportion of individuals, ranging from 15% to 20%, may experience a moderate or severe episode of AP which carry the risk of developing organ failure, whether single or multiple, as well as various significant local consequences such as the development of pancreatic fluid collections (PFC). The revised Atlanta classification has morphologically categorized AP into 2 distinct subtypes: interstitial edematous and necrotizing pancreatitis. Interstitial pancreatitis is characterized by the diffuse or localized enlargement of the pancreas due to inflammatory edema. On the other hand, necrotizing pancreatitis is characterized by necrosis, or tissue death, of pancreatic and peri-pancreatic tissues.^4^ PFC that develop after acute necrotizing pancreatitis (ANP) are classified into 2 broad categories: ANC develop within the first 4 weeks following an episode of ANP and contain a mixture of necrotic material in both solid and liquid forms. ANC usually persist and evolve into WON, which is characterized by the formation of a distinct encapsulating wall. This evolution often occurs approximately 4 weeks following an ANP episode.^12^

The available research on the prevalence of WON is limited, necessitating a need for more precise knowledge regarding the frequency of WON. None of the studies analyzed cases with all types of WON (sterile or infected); rather, the studies analyzed cases of ANP (overestimation) or patients undergoing necrosectomy (underestimation). To date, the incidence of WON remains unknown, as only data regarding the incidence of necrotizing pancreatitis and treated WON are available.^13^

The criteria for performing interventions on PFCs have evolved over time. The previous teaching that cysts larger than 6 cm in diameter, which did not resolve after 6 weeks, should be drained, is no longer accepted. Current guidelines recommend drainage only for symptomatic or infected collections.^14^ Asymptomatic patients should be managed conservatively through regular and careful surveillance.^8^^,^^9^ However, the literature has limited evidence regarding the natural course of asymptomatic patients with WON. A study involving 43 asymptomatic WON patients reported that most of the cases regressed spontaneously or remained asymptomatic during a mean follow-up duration of 7 months. Complications requiring interventional treatment developed in 1/3 of the patients, with the most common complications being infection and spontaneous fistula to the gastrointestinal tract.^7^ Patra et al^15^ found that among 39 patients with WON, 23 (58.9%) experienced spontaneous resolution, 8 (20.5%) required drainage, and 7 patients (17.9%) had persistent cysts but remained asymptomatic during a mean follow-up duration of 6 months.

Currently, there is insufficient information available concerning the criteria for predicting the need for drainage in patients who develop WON and do not require drainage at the time of diagnosis. In a retrospective study, where 23 (27.3%) out of 84 patients who developed WON were managed conservatively, a CT severity index of >7 and a time span exceeding 7 days between the onset of pain and the duration of hospitalization were identified as independent predictors for the necessity of intervention.^16^ In a more recent study (n = 30), a CT severity index >9, a size of WON measuring 127 mm, and a baseline CRP level of 49.5 mg/dl were identified as predictors for the future development of infection in WON.^8^ It has also been reported that extra-pancreatic necrosis was more frequently observed in patients with complete regression of WON during the observation period. On the other hand, mixed (pancreatic and extra-pancreatic) necrosis was much more commonly observed in patients who required interventional treatment for complications related to WON. The more frequent occurrence of extra-pancreatic WON in the group of patients with spontaneous regression of the necrotic collection was attributed to the absence of main pancreatic duct disruption.^17^ The study identified that the need for drainage was correlated with the extent of necrosis and the initial size of WON, which was consistent with the existing literature.

In most asymptomatic cases of WON, the solid necrotic contents gradually liquefy over time. This information was disclosed in a study including 47 patients that prospectively followed up patients with PFC following ANC and analyzed the morphology, including the amount of the solid content, through serial endoscopic ultrasound at 6 weeks, 3 months, and 6 months after the onset of ANC. The follow-up endoscopic ultrasounds at 3 and 6 months revealed a progressive decrease in size as well as reduction in the amount of solid content in the PFC. The mean diameter of the PFC decreased to half of the original size, and over 50% of patients had a PFC with no solid content at 6-month follow-up.^11^ When the size of the WON starts to decrease, it is recommended to monitor the patients every 3 months until complete regression is observed. Complications usually arise during the initial few months, often occurring between 1.63 and 3.5 months, as documented in the literature.^8^^,^^15^ The majority of complications can be effectively managed using minimally invasive techniques, leading to no reported cases of mortality.^12^ In the current study, the majority (80%) of complications occurred within the first 6 months of follow-up. This highlights the significance of closely monitoring patients during the initial months.

The study had several limitations. It was a single-center, retrospective observational study with a limited number of patients. First, due to the retrospective design and the limited number of patients requiring intervention, comprehensive univariate or multivariate analyses could not be performed without compromising statistical validity. Therefore, ROC analysis was applied only to parameters that showed significant differences in descriptive comparison, and this limitation should be considered when interpreting the results. Second, the severity of AP was categorized according to the Revised Atlanta Classification, based on findings during hospitalization. Because initial admission data were not consistently available, predictive severity scores such as Ranson, Bedside index of severity in acute pancreatitis (BISAP) score, or Harmless Acute Pancreatitis Score (HAPS) could not be applied. Third, the study mainly focused on the radiologic characteristics and natural course of WON. Consequently, clinical parameters such as intensive care unit requirement, organ failure, and laboratory inflammatory markers were not analyzed in detail. Similarly, indices such as CRP, systemic inflammatory response syndrome, and CT severity index were not systematically recorded for all patients and therefore could not be included in the analysis. Finally, because this was a single-center study with a relatively small sample size, the findings may not be generalizable to all patient populations. Future prospective, multicenter studies integrating both clinical and radiologic variables are needed to validate these observations and to better identify predictors of intervention in patients with WON. On the other hand, the study also had several strengths, including consistent clinical and radiological follow-ups, precise classification of fluid collections, exclusion of patients with chronic pancreatitis, a longer follow-up duration (range: 17-51 months) compared to previous studies (range: 6-32 months) and the utilization of a validated scoring system for assessing severity. Furthermore, the literature comprises only a limited number of studies on the natural history of WON, and the study significantly contributed to the existing body of research.

In conclusion, it is crucial to note that the natural course of WON is unpredictable, and outcomes may substantially differ among individuals. Therefore, the management of WON usually includes close monitoring of the patient’s clinical status, conducting imaging studies to assess the collection’s size and changes, and considering intervention if complications develop or the patient fails to improve. The initial characteristics of WON (e.g., extent of necrosis, size, etc.) may aid in determining the necessity for drainage during the follow up of asymptomatic patients. Further long-term follow-up studies, involving larger patient cohorts, are required to investigate the issue.

## Figures and Tables

**Table 1.** The Baseline Demographic, Clinical, Radiologic Features and the Therapeutic Interventions of all Patients with WON

	Results (n = 46)
Age, years (mean ± SD)	58.3 ± 13.8
Gender, n (%) Male Female	26 (56.5) 20 (43.5)
Etiology, n (%) Biliary Alcohol Hypertriglyceridemia Drug Idiopathic	26 (56.5) 7 (15.2) 3 (6.5) 1 (2.2) 8 (17.4)
Severity of AP Moderate Severe	35 (76.1) 11 (23.9)
Comorbidity, n (%) Hypertension Diabetes mellitus Coronary artery disease Others None	17 (37.0) 4 (8.7) 8 (17.3) 8 (17.3) 20 (43.5)
Number of WON, n (%) 1 >1	24 (52.2) 22 (47.8)
Location of WON, n (%) Pancreatic Extra-pancreatic Combined	38 (82.6) 7 (15.2) 1 (2.2)
Intra-pancreatic location of WON, n (%) Multiple Head Corpus Uncinate	30 (76.9) 3 (7.6) 5 (12.8) 1 (2.5)
Size of WON, mm (mean ± SD)	107.2 ± 48.5
Wall thickness of WON, mm (mean ± SD)	3.1 ± 1.7
Extent of necrosis in WON, % (mean ± SD)	52.7 ± 31.6
Patients requiring therapeutic intervention at initial admission, n (%)*	11 (26.1)
Indications for drainage, n (%) Abdominal pain Fever Nausea-Vomiting Pain + Fever	3 (27.3) 3 (27.3) 2 (18.1) 3 (27.3)
Drainage methods, n (%)** Endoscopic Percutaneous Surgery	3 (42.8) 2 (28.5) 2 (28.5)

AP, acute pancreatitis, WON, walled-off necrosis.

*Four patients were excluded from the study because of insufficient follow-up data.

**Four patients died without any intervention, despite the indication for drainage.

**Table 2.** The Demographic, Clinical, and Radiologic Features of the Study Group

	Results (n = 31)
Age, years (mean ± SD)	59.3 ± 14.2
Gender, n (%) Male Female	17 (54.8) 14 (45.2)
Etiology, n (%) Biliary Alcohol Hypertriglyceridemia Idiopathic	19 (61.3) 5 (16.1) 2 (6.5) 5 (16.1)
Severity of AP Moderate Severe	26 (83.9) 5 (16.1)
Comorbidity, n (%) Yes None	16 (51.6) 15 (48.4)
Number of WON, n (%) 1 >1	16 (51.6) 15 (47.8)
Location of WON, n (%) Pancreatic Extra-pancreatic Combined	24 (77.4) 6 (19.4) 1 (3.2)
Intra-pancreatic location of WON, n (%) Multiple Head Corpus	18 (75) 2 (8.3) 4 (16.7)
Size of WON, mm (mean ± SD)	101.1 ± 48
Wall thickness of WON, mm (mean ± SD)	2.8 ± 0.9
Extent of necrosis in WON, % (mean ± SD)	57.1 ± 24.0

AP, acute pancreatitis; WON, walled-off necrosis.

**Table 3.** The Therapeutic Interventions in the Study Population During the Long-Term Follow-up

	Results (n = 31)
Mean follow-up period, months (mean ± SD)	25.6 ± 17.3
Patients requiring therapeutic intervention, n (%)	10 (32.3)
Time for therapeutic intervention, days (mean ± SD)	124.9 ± 189.5
<3 months, n (%) 3-6 months, n (%) 6-12 months, n (%) >12 months, n (%)	6 (60) 2 (20) 1 (10) 1 (10)
Indications for drainage, n (%) Abdominal pain, vomiting Fever	6 (60) 4 (40)
Drainage methods, n (%) Endoscopic Percutaneous Surgical Splenic artery embolization+Surgical	5 (50) 3 (30) 1 (10) 1(10)

AP, acute pancreatitis; WON, walled-off necrosis.

**Table 4.** Comparison of the Patients Requiring Drainage and those not Requiring Drainage

	Drainage (+) n: 10	Drainage (−) n: 21	*P*
Age, years (mean ± SD)	60.3 ± 15.4	58.9 ± 14	.81
Female, n (%)	4 (40)	10 (47.6)	1
Etiology, n (%) Biliary Non-biliary	9 (90) 1 (10)	10 (47.6) 11 (52.4)	**<.05**
Severity of AP, n (%) Moderate Severe	8 (80) 2 (20)	18 (85.7) 3 (14.3)	1
Location of WON, n(%) Pancreatic Extrapancreatic Combined	9 (90) 1 (10)	15 (71.4) 5 (23.8) 1 (4.8)	.4
Number of WON, n(%) 1 >1	5 (50) 5 (50)	11 (52.4) 10 (47.6)	1
Size of WON, mm (mean ± SD)	125.9 ± 46.6	89.3 ± 45	***<*.05**
Wall thickness of WON, mm (mean ± SD)	2.9 ± 0.7	2.7 ± 0.9	.86
Extent of necrosis in WON, % (mean ± SD)	76.1 ± 16.3	48.4 ± 21.2	**.004**

AP, acute pancreatitis; WON, walled-off necrosis.

## Data Availability

The data that support the findings of this study are available on request from the corresponding author.
